# Allochthonous carbon primarily of marine origin in Irish saltmarshes: novel insights from bacteriohopanepolyol biomarkers

**DOI:** 10.1093/sumbio/qvaf012

**Published:** 2025-07-17

**Authors:** Saule Akhmetkaliyeva, Robert B Sparkes, Eliza Fairchild, Ragna Hoogenboom, Grace M Cott

**Affiliations:** School of Biology and Environmental Science, University College Dublin, Dublin D04 V1W8, Ireland; Department of Natural Sciences, Manchester Metropolitan University, Manchester M1 5GD, United Kingdom; School of Biology and Environmental Science, University College Dublin, Dublin D04 V1W8, Ireland; School of Biology and Environmental Science, University College Dublin, Dublin D04 V1W8, Ireland; School of Biology and Environmental Science, University College Dublin, Dublin D04 V1W8, Ireland

**Keywords:** saltmarsh, blue carbon, carbon source, bacteriohopanepolyol, bacteria, allochthonous, loss-on-ignition

## Abstract

Coastal blue carbon ecosystems (BCEs) are among the most effective carbon (C) sinks, yet Irish saltmarshes, covering 4000–6500 hectares, remain understudied in terms of C sources. Understanding saltmarshes’ connectivity to marine and terrestrial sources, and the provenance of allochthonous (ex-situ-produced) carbon, is essential for assessing their long-term C sequestration potential and management. This study analysed organic carbon (OC) and bacteriohopanepolyol biomarkers (BHPs), microbial lipids that trace sedimentary OC sources, in sediment cores from Derrymore Island (DI) and North Bull Island (NBI). OC concentrations varied down-core, ranging from 0.26–26.68 wt% at DI, 0.44–12.19 wt% in NBI’s North Lagoon, and 0.07–26.45 wt% in its South Lagoon. The *R*_soil_ index values (0.02–0.24 at DI, 0.01–0.06 at NBI) indicate a predominantly marine OC origin, though soil-specific BHPs were present, with higher soil marker concentrations in deeper, older DI sediments. This suggests saltmarshes may gradually transition toward terrestrial characteristics over time. This study enhances our understanding of OC sequestration in two Irish saltmarshes, highlighting their dynamic nature. The allochthonous nature of sequestered sedimentary OC further underscores the importance of sustained tidal influence and connectivity with the wider marine environment. Further research into labile OC sources is recommended to enhance carbon assessments in these ecosystems.

**Sustainability Statement**

This manuscript investigates carbon sources in Irish saltmarshes, directly contributing to the UN Sustainable Development Goals (SDGs) 13 (climate action), 14 (life below water), and 15 (life on land). By identifying the origins of organic carbon, the study deepens our understanding of their critical role in mitigating climate change (SDG 13). The findings underscore the importance of tidal connectivity in maintaining saltmarsh carbon storage, which informs strategies for sustainable coastal management and conservation (SDG 14). Additionally, the research provides insights into the gradual transition of saltmarshes to more terrestrial environments over time, informing land-use policies and ecosystem restoration efforts (SDG 15). Ultimately, this research supports global efforts to preserve blue carbon ecosystems and their essential role in long-term carbon sequestration.

## Introduction

Human-induced climate change, driven by the increased release of greenhouse gases, such as carbon dioxide (CO_2_) and methane (CH_4_), is a critical global issue. Global surface temperatures have risen by 1.1°C in the period of 2011–2020 compared to 1850–1900 (IPCC 2023). Natural ecosystems, including terrestrial, coastal, and marine systems, play a vital role in organic carbon (OC) absorption and storage. In recent years, research on ‘blue carbon’ ecosystems (BCEs), such as saltmarshes, seagrass meadows, and mangrove forests, has gained momentum due to their ability to sequester OC (Duarte et al. 2005, McLeod et al. 2011, Alongi 2012, Rogers et al. 2019). These coastal habitats can sequester OC for centuries to millennia (Duarte et al. 2005). Despite their relatively small area, BCEs store OC at rates (g C m^–2^ yr^–1^) an order of magnitude higher than terrestrial ecosystems, making them a key tool for climate mitigation and adaptation (McLeod et al. 2011, Were et al. 2019). Moreover, BCEs provide critical ecosystem services, including wildlife habitats (Minello et al. 2003, Sutton-Grier and Sandifer 2019), shoreline protection from erosion, and buffering against storm surges (Möller et al. 2014, Unsworth and Butterworth 2021).

Carbon sequestration in BCEs is influenced by both autochthonous and allochthonous OC sources (Kennedy et al. 2010, Komada et al. 2022, Li 2025). Autochthonous OC is captured through *in situ* plant productivity and the accumulation of organic matter (OM), such as belowground roots, rhizomes, and aboveground vegetation (Saintilan et al. 2013). Allochthonous OC is transported to the marsh through the trapping of suspended particulate OM and sediment from terrestrial and marine sources (Saintilan et al. 2013). The anoxic conditions in saltmarshes further promote efficient OC storage (Duarte et al. 2013, Kirwan and Megonigal 2013). Since saltmarshes form a transitional zone between marine and terrestrial environments, identifying the origins of OC and understanding the relative contributions of these OC sources—termed ‘carbon provenance’—is critical, as these sources differ in stability and decomposition rates, directly influencing long-term OC sequestration potential. This knowledge informs climate change mitigation strategies by identifying which habitats are most effective for carbon storage and should be prioritized for conservation or restoration (Saintilan et al. 2013, Komada et al. 2022). Advanced analytical techniques, such as organic biomarkers, could provide valuable insights into OC provenance in these ecosystems.

Organic biomarkers, such as bacteriohopanepolyols (BHPs), are useful for identifying bacterial communities and the origins of OC. Bacteriohopanepolyols, bacterial membrane lipids produced by various bacterial groups, are effective biomarkers due to their structural diversity, which can provide taxonomic and physiological insights (Kusch and Rush 2022) (Supplementary Fig. S1). Soil-specific nucleoside BHPs, like 30-(5′-adenosyl)hopane, help distinguish between plant-derived and soil-derived OM and often indicate terrestrial inputs when found in sediments (Zhu et al. 2011, Doğrul Selver et al. 2012), although some studies suggest they can also be produced at low levels by non-terrestrial bacteria under certain redox conditions, such as in marine oxygen-deficient zones (Kusch et al. 2021). Their presence in ancient sediments demonstrates their resistance to degradation over time (van Dongen et al. 2006). The *R*_soil_ index, when used alongside BHPs, further helps trace soil-derived OC by comparing concentrations of soil-marker BHPs to bacteriohopanetetrol (BHT), a common pseudo-marine BHP. Values near zero indicate marine OC, while those between 0.5 and 0.8 suggest primarily terrestrial sources (Zhu et al. 2011). While we acknowledge that saltmarshes host indigenous microbial communities capable of producing BHPs, our focus is on the provenance of OC in accreted sediments, where both local and external inputs are integrated. Saltmarshes are treated as transitional systems in this study, and BHP distributions are interpreted in that context. This is the first study to apply BHPs to assess OC sedimentary sources in saltmarsh habitats.

Ireland hosts ∼4000–6500 ha of saltmarsh habitats (Perrin 2020, Burke 2024), which are estimated to store up to 950 000 Mg OC (Burke 2024). Despite their ecological and climate significance, there are currently no studies on the source of OC in BCEs in Ireland, including saltmarshes.

The primary objective of this study is to investigate sedimentary OC sources in Irish saltmarshes using BHPs. This research seeks to improve understanding of the factors influencing OC sequestration and its potential implications for climate change mitigation. Specifically, the following questions are addressed: (i) What are the BHP signatures in Irish saltmarshes? (ii) Are the sources of sedimentary OC predominantly marine or terrestrial in these transitional systems? (iii) What factors drive the variation in BHP signatures across different saltmarshes in Ireland? (iv) Which sources of OC should be considered when evaluating carbon sequestration potential? Finally, the study also aims to contribute to the understanding of the temporal development and changes in saltmarsh ecosystems.

## Study area

**Figure 1.**
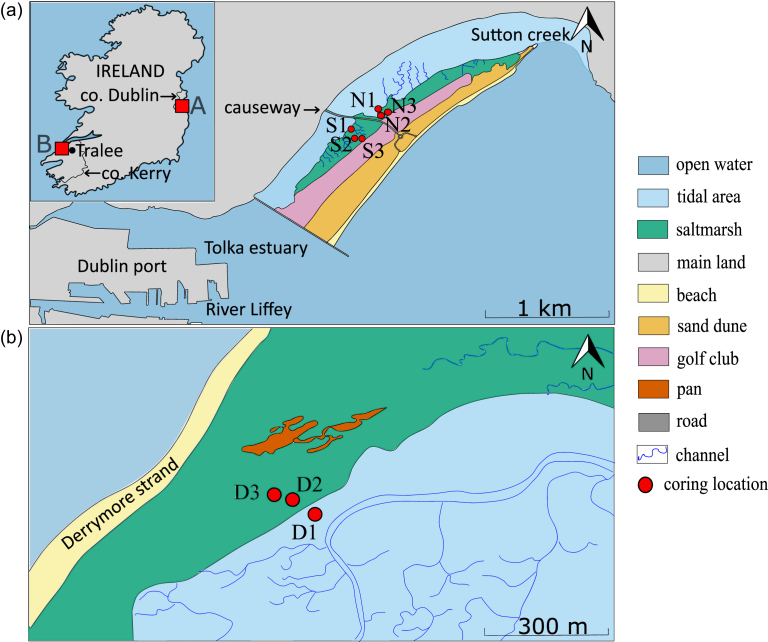
Sampling sites at: (a) North (N1, N2, and N3) and south (S1, S2, and S3) lagoons at the NBI, E Ireland and (b) DI (D1, D2, and D3), SW Ireland. Inset shows location of study areas within Ireland.

The carbon provenance study was conducted across two sites in Ireland: North Bull Island (NBI) (53°22′19“N, 6°9′11″W) and Derrymore Island (DI) (52°15′9“N, 9°49′58″W) saltmarshes (Fig. 1).

### North bull island

NBI is a coastal sand spit that extends 5 km in a northeastward direction from the northern boundary of Dublin Port in County Dublin. The island emerged ∼200 years ago as a result of port development, with sediment deposition from the River Liffey leading to the formation of a saltmarsh covering a total area of ∼120 ha (Burke et al. 2022). In 1964, a causeway was constructed across the island, dividing the saltmarsh into two lagoons: the south lagoon (NBIS) and the north lagoon (NBIN) (Grey et al. 2021). The hydrological regimes of these lagoons differ, with the NBIS primarily receiving seawater from the Tolka estuary, while the NBIN is fed by a mixture of seawater and freshwater entering through Sutton Creek and influenced by the River Liffey plume (Grey et al. 2021).

The climate in Dublin, where NBI is situated, is temperate and characterized by mild winters and cool summers, with limited seasonal temperature variation. In 1942–2022, the mean daily air temperature through the year ranged from 6.1°C to 13°C, and the mean monthly precipitation was 62.9 mm, based on data from the Dublin Airport station (1942–2022, Dublin Airport station, Met Éireann).

### Derrymore island

DI saltmarsh is a natural tidal estuarine marsh located east of Derrymore Strand and west of Tralee town in County Kerry, southwest Ireland. The saltmarsh is subject to regular tidal inundation, which brings seawater over its surface during high tides. The estuarine area, including the saltmarsh, has been designated a special protection area due to its ecological and conservation significance, owing to the biodiversity it supports (wintering waders and wildfowl such as whooper swans, golden plover, and bar-tailed godwit) (NPWS 2021). The island has historically been used for cattle grazing, a practice that continues today.

The saltmarsh at DI is much older compared to NBI. Sediments in the pioneering and lower-marsh zones have been dated to ∼100 years old within the top 20 cm (Burke 2024). The sediment depth reached 49, 57, and 39 cm in lower-, mid- and upper-marsh areas, respectively. While the exact age of the saltmarsh itself remains uncertain, upper marsh zones tend to have older sediments compared to lower-marsh zones due to differences in flooding frequency and sedimentation rates (Cornacchia et al. 2024). Therefore, it is anticipated that the sediments in the mid- and upper-marsh zones are much older (several centuries to over a 1000 years), reflecting a longer history of sediment accumulation and marsh development.

The climate in this region of southwest Ireland is wetter than that of Dublin. In 1981–2022, the mean daily air temperature ranged from 7.1°C to 14.2°C, with a mean monthly precipitation of 97.5 mm (1981–2022, Ardfert (Liscahane) station, Met Éireann).

## Methods

### Field sampling

In April 2023, nine soil cores were collected using a 6 cm diameter gouge auger (Van Walt, Haslemere, UK), following the protocol outlined in the Coastal Blue Carbon manual (Howard 2014). Coring was conducted until the refusal point was reached, with no evidence of sediment compaction observed during the process. Three cores were extracted from NBIN, three from NBIS, and three from DI. Cores were taken from the lower-, mid-, and upper-marsh zones to represent different sub-habitats within the saltmarsh.

To investigate carbon accumulation over time, each core was sectioned into depth intervals of 0–5 cm, 5–15 cm, 15–25 cm, 25–35 cm, 35–50 cm, and 50–100 cm (or until the refusal depth). Further, from each section, the central 5 cm subsample was carefully extracted using a stainless-steel spatula and placed in a pre-combusted aluminium foil packet (450°C for 4 h). Sub-samples were transported in a cool bag and stored at 4°C to maintain sample integrity until further analysis. Sample descriptions are provided in Table 1.

**Table 1.** Sampling locations, subsample intervals, organic carbon concentration (EA), loss-on-ignition (LoI) following Howard (2014) and Burke et al. (2022) protocols and C/N ratio of downcore samples from DI, NBI North Lagoon, and NBI South Lagoon saltmarshes.

Sample name	Saltmarsh	Marsh zone	Latitude, longitude	Depth, top (cm)	Depth, bottom (cm)	OC_EA_ (wt%)	LoI_500 (%)	LoI_450 (%)	C/N
D1A	Derrymore	lower	52°15′08“N 9°49′56″W	0	5	5.35	13.17	14.00	10
D1B				5	15	4.65	11.84	12.00	9
D1C				15	25	2.62	7.94	9.00	9
D1D				25	35	1.08	2.34	3.00	11
D1E				35	49	0.26	1.53	2.00	4
D2A	Derrymore	mid	52°15′09“N 9°49′57″W	0	5	10.11	24.36	27.00	11
D2B				5	15	8.79	19.89	21.00	10
D2C				15	25	4.69	11.17	12.00	11
D2D				25	35	1.96	5.50	5.00	9
D2E				35	50	2.01	5.31	6.00	9
D2F				50	57	3.67	8.80	8.00	12
D3A	Derrymore	upper	52°15′10“N 9°49′59″W	0	5	26.68	51.75	71.00	14
D3B				5	15	8.18	22.04	23.00	13
D3C				15	25	2.25	4.67	7.00	13
D3D				25	35	0.60	1.43	2.00	9
D3E				35	39	0.28	0.90	1.00	6
N1A	North Lagoon, NBI	lower	53°22′24“N 06°09′07″W	0	5	4.82	12.73	10.00	9
N1B				5	15	3.57	9.57	6.00	9
N1C				15	25	2.28	6.15	4.00	10
N1D				25	35	0.84	2.45	2.00	10
N1E				35	40	0.49	1.72	1.00	9
N2A	North Lagoon, NBI	mid	53°22′22“N 06°09′05″W	0	5	6.58	6.50	14.00	10
N2B				5	15	5.40	13.64	12.00	11
N2C				15	25	6.04	15.29	15.00	11
N2D				25	35	5.65	11.11	9.00	12
N2E				35	50	0.64	1.47	2.00	9
N2F				50	57	0.63	1.72	1.00	9
N3A	North Lagoon, NBI	high	53°22′22“N 06°09′07″W	0	5	12.19	31.12	27.00	12
N3B				5	15	3.18	9.16	7.00	12
N3C				15	25	2.63	8.49	4.00	13
N3D				25	35	7.48	15.56	7.00	11
N3E				35	50	2.04	6.03	3.00	9
N3F				50	52	0.44	1.81	1.00	6
S1A	South Lagoon, NBI	lower	53°22′17“N 06°09′29″W	0	5	1.90	6.25	4.00	8
S1B				5	15	2.36	7.39	6.00	10
S1C				15	25	2.25	6.24	5.00	10
S1D				25	35	0.45	0.00	1.00	9
S2A	South Lagoon, NBI	mid	53°22′10“N 06°09′24″W	0	5	18.29	39.74	32.00	11
S2B				5	15	17.90	37.98	35.00	11
S2C				15	25	11.65	27.96	29.00	11
S2D				25	35	3.13	9.96	9.00	9
S2E				35	50	0.16	0.89	0.00	4
S2F				50	59	0.07	0.75	1.00	2
S3A	South Lagoon, NBI	upper	53°22′09“N 06°09′19″W	0	5	26.45	60.28	59.00	13
S3B				5	15	5.58	14.26	15.00	12
S3C				15	25	4.76	13.69	14.00	10
S3D				25	29	0.58	2.27	1.00	8

### OC analysis

The soil samples were dried following the Coastal Blue Carbon manual at 60°C for 72 h (Howard 2014), then sieved through a 2 mm mesh and homogenized before further analysis. OC was removed by ashing the samples. LoI analysis was performed twice: first at 450°C for 4 h (LoI_450_), as described by Burke et al. (2022), and then at 500°C for 12 h (LoI_500_), following the recommended protocol in Howard (2014). OC concentrations derived from LoI_500_ were calculated using the following equation from Craft et al. (1991):


(1) \begin{eqnarray*}
{\mathrm{O}}{{{\mathrm{C}}}_{{\mathrm{LoI}}}}{\mathrm{ = (0}}{\mathrm{.40 \pm 0}}{\mathrm{.01) \times LoI + (0}}{\mathrm{.0025 \pm 0}}{\mathrm{.0003) \times Lo}}{{{\mathrm{I}}}^{\mathrm{2}}}.\\
\end{eqnarray*}

Total organic carbon (TOC) concentration was determined using a Vario EL Cube (Elementar) elemental analyser. Approximately 0.2 g of the dried samples were analysed for total carbon (TC), while the LoI_500_ samples were analysed for total inorganic carbon (TIC). The concentration of TOC was determined by subtracting TIC from TC. Approximately 0.15 g of EDTA (Ethylenediaminetetra-acetic acid) 502–092 was used as a calibration standard and ∼0.2 g of soil standard (clay) (elemental microanalysis) as a certified reference material to ensure data accuracy and precision.

### Organic biomarker analysis

#### Biomarker extraction and BHP separation

Soil samples were extracted using a modified Bligh–Dyer method, as detailed in Akhmetkaliyeva et al. (2025). Approximately five grams of soil underwent ultrasonic extraction for 10 min at 40°C using 19 ml of a methanol (MeOH), dichloromethane (DCM), and de-ionized water (DI) mixture in a 2:1:0.8 (v:v:v) ratio. The supernatant was separated via centrifugation at 2 500 RPM for five minutes. This process was repeated twice with the remaining sample. To isolate the organic phase, the supernatants were combined with DCM and DI water in a final ratio of 1:1:0.9 (v:v:v). and evaporated under a nitrogen stream. The total lipid extract (TLE) was recovered using a DCM and MeOH mixture (2:1, v:v) and divided into three aliquots.

NH_2_ solid-phase extraction (SPE) cartridges (Supelclean™ LC-NH2 SPE Tube, Merck Life Science UK Limited) were used to purify BHP compounds from the TLE. Each cartridge was pre-conditioned with 6 ml of hexane before one TLE aliquot was redissolved in 200 µl of DCM and loaded onto the cartridge. Non-polar and acidic compounds were removed by washing with 6 ml of a diethyl ether and acetic acid mixture (98:2, v:v). Polar compounds, including BHPs, were eluted using 10 ml of methanol and dried under a nitrogen stream.

An internal standard, 200 µl of 5α-pregnane-3β,20β-diol (Tokyo Chemical Industry UK Ltd.), was added to the BHP fraction. The fraction was acetylated overnight with a pyridine and acetic anhydride mixture (1:1, v:v), followed by evaporation under a nitrogen stream. The sample was redissolved in a propan-2-ol and MeOH mixture (60:40, v:v), filtered through a 0.2 µm PTFE syringe filter, and dried. Finally, the sample was dissolved again in a MeOH and propan-2-ol mixture (60:40, v:v) and analysed by high-performance liquid chromatography/atmospheric pressure chemical ionization-mass spectrometry (HPLC/APCI-MS).

#### HPLC-Q-TOF-MS analysis

BHPs were separated, identified and quantified using an Agilent 1200 series HPLC with a 15 cm C_18_ column coupled to an Agilent Technologies 6540 UHD Accurate-Mass Q-TOF mass spectrometer, equipped with a positive ion APCI source (Cooke et al. 2008a, Pytlak et al. 2021) of MeOH: DI water (90:10, v:v) for solvent A and MeOH: propan-2-ol: DI water (59:40:1, v:v:v) for solvent B. The solvent gradient initiated at 100% A (MeOH: DI water, 90:10, v:v), transitioned to 100% B (MeOH: propan-2-ol: DI water, 59:40:1, v:v:v) over 25 min, and was held for 15 min. After BHP separation, to remove contaminants, the column was backflushed and re-equilibrated with 100% A. APCI conditions included a drying gas flow rate of 8 l/min at 300°C, a nebulizer pressure of 35 psig, a vaporizer temperature of 400°C, a capillary voltage of 3.5 kV, and a corona current of 8 μA.

The structures of BHPs (Fig. S1) were identified using MassHunter Acquisition Software (Agilent, USA), by comparison of absolute and relative retention times, major ions, and MS^2^ ions to previously published spectra (Cooke et al. 2008a). The semi-quantitative concentration of BHP compounds was calculated comparing the base peak area of the internal standard (5α-pregnane-3β,20β-diol, Tokyo Chemical Industry UK Ltd.) at m/z 345 with the corresponding area of individual BHPs and applying standard scaling factors for an ion-trap MS (Cooke et al. 2008a). As our analysis was performed on a Q-TOF-MS system, some deviation in response may occur, potentially introducing minor error in quantification. Average relative standard deviation of duplicate injections was 2% (±0.40 µg/g sediment; 1 standard deviation) for BHT and 8% (±0.01 µg/g sediment; 1 standard deviation) for adenosylhopane.

The BHPs were categorized into five classifications: BHTs, amino, sugar, aminosugar, and soil marker BHPs (specified in Table S1). The BHP-based R_soil_ index was calculated using equation (2) (Zhu et al. 2011):


(2) \begin{eqnarray*}
{{R}_{\textit{soil}}} = \frac{{\textit{soil}\ \textit{marker}\ \textit{BHPs}}}{{\textit{soil}\ \textit{marker}\ \textit{BHPs} + BHT}}.
\end{eqnarray*}

## Results

### OC measurements

#### Loss-on-ignition versus elemental analysis

The content of OC determined via elemental analysis (OC_EA_) was highest in samples from DI, followed by NBIS, and lowest in NBIN, ranging from 0.26 to 26.68 wt%, 0.07 to 26.45 wt%, and 0.44 to 12.19 wt%, respectively (Table 1). OC concentrations derived from LoI (500°C for 12 h) were calculated for the same samples using the equation 1 from Craft et al. (1991) that models the relationship between OC_LoI_ (OC determined via LoI) and LoI in marsh environments. While the OC_LoI_ values were comparable, they consistently overestimated OC content across all sites. On average, the OC_LoI_ values were 16% higher at NBIS, 8% at DI, and 5% at NBIN than the OC_EA_ values (Table 1).

LoI_500_ (500°C for 12 h) and LoI_450_ (450°C for 4 h) gave similar results overall (Table 1). Although the two LoI iterations were closely correlated (3.37% uncertainty, *R*² = 0.95; Fig. S2), they were significantly different (*P*-value <0.05). Greater variability was observed in samples with higher OM content. Despite being a cooler and shorter method, there were some samples where LoI_450_ lost more mass than LoI_500_.

The relationship between OC and LoI was assessed for each saltmarsh using both linear and polynomial regression models (Fig. S3), resulting in site-specific equations in Table 2.

- At DI, the polynomial model (4) provided a near-perfect fit (*R*^2^ = 1.00, *P*-value <0.05) for estimating OC from LoI, outperforming the linear model (3) (*R*^2^ = 0.98, *P*-value <0.05).
- For NBIN, the linear model (5) achieved a higher *R*^2^ value (0.95, *P*-value <0.05) compared to the polynomial model (6) (*R*^2^ = 0.88, *P*-value <0.05).
- At NBIS, both the linear (7) (*R*^2^ = 0.99, *P*-value <0.05) and polynomial (8) (*R*^2^ = 0.99, *P*-value <0.05) models exhibited strong fits.

**Table 2.** Site specific OC-LoI linear and polynomial equations.

Saltmarsh	Equation	*No*	*R* ^2^
Derrymore island	OC = 0.4663 x LoI	(3)	0.98
	OC = 0.3482 x LoI + 0.0032 x LoI^2^	(4)	1.00
NBI, North lagoon	OC = 0.4119 x LoI	(5)	0.95
	OC = 0.4499 x LoI–0.0019 x LoI^2^	(6)	0.88
NBI, South lagoon	OC = 0.4399 x LoI	(7)	0.99
	OC = 0.3999 x LOI + 0.0009 x LoI^2^	(8)	0.99

#### Site specific OC_EA_ measurements

**Figure 2.**
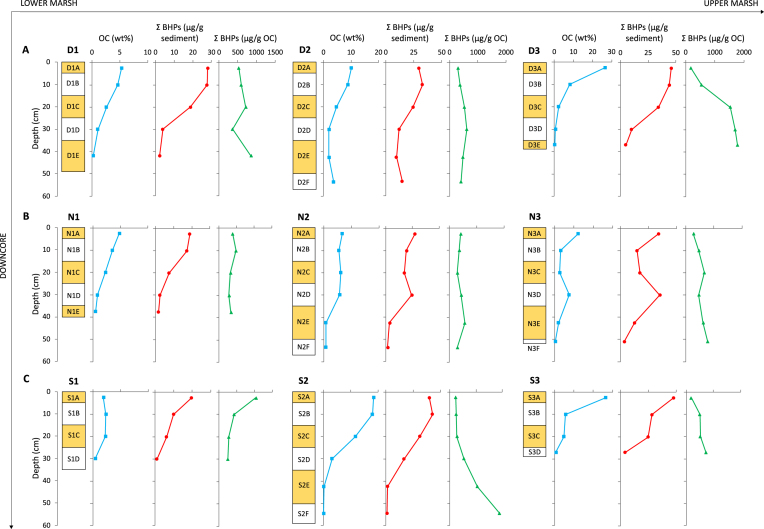
Downcore organic carbon (EA) (wt%), total absolute BHP (µg/g sediment) and total relative BHP (µg/g OC) concentrations across saltmarsh cores from lower-, mid- and upper-marsh areas at (a) DI, (b) NBI, north lagoon, and (c) NBI, south lagoon. The depths are representative of the average of the subsample.

Site-specific differences in OC_EA_ concentrations were observed: NBIS showed elevated OC_EA_ levels, NBIN had the lowest, and DI exhibited high OC_EA_ values (Table 1). OC values decreased with depth across all three sites (Fig. 2, Table 1). For example, in cores from DI, OC_EA_ concentrations on average dropped by 65% around 20 cm and stabilized below 30 cm. The concentrations of OC_EA_ also varied along transects, increasing from the lower to the upper-marsh zones (Fig. 2). This pattern was most pronounced at the top of the core across all sites and became less distinct with depth.

C:N ratios ranged from 1.8 to 14.5 (Table 1). The lowest C:N values (e.g. 1.8) were observed in the deepest sediments at NBI, whereas higher C:N ratios were generally associated with surface sediments.

### Total BHPs

This study detected BHPs in all analysed samples, marking a novel application of BHP analysis in saltmarsh environments. Since BHPs represent a fraction of OC, their concentrations can be normalized either to sediment weight (absolute BHP concentration, e.g. per gram of sediment) or to OC content (relative BHP concentration, e.g. per gram of OC). Total BHP concentration reflects the overall abundance of BHPs in the sediment.

#### Absolute total BHPs

Absolute total BHP concentrations varied significantly across the three study sites. Total BHP concentrations ranged from 2 to 46 µg/g sediment at DI, 2 to 35 µg/g sediment at NBIN, and 1 to 47 µg/g sediment at NBIS (Table 3). Higher BHP concentrations in DI and NBIS compared to NBIN mirrored the trends in OC_EA_ content across the sites, with a strong correlation between BHP and OC concentrations (*R*^2^ = 0.99, *P*-value <0.05).

**Table 3.** BHP compound group concentrations (µg/g OC), total relative (µg/g OC) and total absolute concentration of BHPs (µg/g sediment), and *R*_soil_ values in Irish saltmarshes. BDL—below detection limit.

Sample name		BHP compound groups					Σ BHPs (µg/g OC)	Σ BHPs (µg/g sediment)	*R*_soil_
	Anhydro	BHTs	Amino	Sugar	Aminosugar	Soil			
D1A	0.88	466	22	4	12	20	525	28	0.04
D1B	0.78	507	33	6	19	29	594	28	0.06
D1C	1.73	586	47	10	22	46	713	19	0.08
D1D	2.22	278	28	5	14	29	357	4	0.11
D1E	7.83	689	66	10	29	54	855	2	0.08
D2A	BDL	251	23	BDL	11	6	292	30	0.03
D2B	BDL	292	59	BDL	16	6	373	33	0.02
D2C	0.43	451	41	BDL	14	15	522	24	0.04
D2D	1.09	512	38	BDL	13	45	608	12	0.09
D2E	0.79	372	28	BDL	26	47	473	10	0.12
D2F	1.04	285	33	BDL	24	61	405	15	0.19
D3A	BDL	115	33	BDL	15	10	172	46	0.08
D3B	BDL	290	86	BDL	92	72	540	44	0.22
D3C	2.92	884	289	BDL	107	244	1 528	34	0.23
D3D	2.01	898	457	3	134	204	1 698	10	0.20
D3E	1.88	788	583	BDL	167	234	1 774	5	0.24
N1A	0.68	335	39	BDL	13	3	391	19	0.01
N1B	1.27	417	45	BDL	18	4	485	17	0.01
N1C	1.27	276	29	BDL	16	5	328	7	0.02
N1D	2.24	226	32	BDL	19	9	287	2	0.04
N1E	3.40	264	38	BDL	26	8	339	2	0.03
N2A	0.73	311	63	BDL	36	2	412	27	0.01
N2B	0.83	299	39	BDL	14	3	356	19	0.01
N2C	0.87	237	37	BDL	10	3	288	17	0.01
N2D	0.58	346	56	BDL	23	2	428	24	0.01
N2E	1.16	389	124	BDL	38	8	560	4	0.02
N2F	1.91	201	45	BDL	32	11	291	2	0.06
N3A	BDL	211	41	BDL	21	4	276	34	0.02
N3B	BDL	395	36	BDL	26	9	466	15	0.03
N3C	1.08	558	53	BDL	30	18	659	17	0.03
N3D	BDL	385	57	BDL	19	6	466	35	0.02
N3E	BDL	555	41	BDL	15	7	619	13	0.01
N3F	BDL	703	42	BDL	20	13	778	3	0.02
S1A	BDL	735	206	BDL	88	6	1 036	20	0.01
S1B	0.95	361	49	BDL	10	3	424	10	0.01
S1C	1.75	248	22	BDL	5	4	279	6	0.02
S1D	2.75	211	26	BDL	5	3	248	1	0.02
S2A	BDL	129	43	BDL	44	2	218	40	0.01
S2B	0.11	143	52	BDL	35	8	238	43	0.06
S2C	0.31	176	54	BDL	26	9	266	31	0.06
S2D	0.78	417	83	BDL	27	8	536	17	0.02
S2E	5.95	860	124	BDL	48	15	1 052	2	0.02
S2F	15.25	1597	200	BDL	80	12	1 904	1	0.01
S3A	BDL	119	31	BDL	26	3	179	47	0.03
S3B	BDL	287	136	BDL	63	18	504	28	0.06
S3C	0.96	347	130	BDL	32	11	520	25	0.03
S3D	1.69	524	140	BDL	39	21	726	4	0.04

Across all nine cores, BHP distributions generally followed the same depth-related trends as OC, decreasing with depth (Fig. 2). However, the decline in BHP concentrations was more gradual than the corresponding decrease in OC_EA_. At DI, BHP concentrations stabilized below 30 cm. In contrast, cores from NBIN or NBIS showed continued variation in BHP concentrations with depth. An upmarsh gradient in BHP concentrations was also observed, with values increasing from the lower to the upper-marsh zones (Fig. 2).

#### Relative total BHPs

The relative concentration of total BHPs ranged from 172 to 1788 µg/g OC at DI, 281 to 786 µg/g OC at NBIN, and 179 to 1931 µg/g OC at NBIS (Table 3). The concentrations normalized by OC exhibited a clear upmarsh increase across all sites (Fig. 2). In the lower marsh cores (N1 and S1), relative total BHP concentrations generally decreased downcore, while D1 had a varying trend downcore. In contrast, upper-marsh cores (D3, N3, and S3) displayed a different pattern, with relative total BHP concentrations mirroring OC_EA_ distributions and increasing downcore. Mid-marsh cores (D2, N2, and S2) showed more variable downcore trends. In the top 30 cm, relative total BHP concentrations aligned with OC_EA_ patterns, while below this depth, concentrations decreased inconsistently.

Samples from DI and NBIS exhibited similarly high relative total BHP concentrations (Table 3), but DI samples displayed greater structural diversity in BHPs (Fig. S1, Table S1, Table S3). In contrast, although NBIS was only slightly older than NBIN, NBIN samples had comparable structural diversity to NBIS, but their relative total BHP concentrations were lower (Tables S2 and S3). Surface samples from the upper marsh cores at DI and NBIN, and mid- and upper-marsh cores at NBIS had lower relative total BHP concentrations compared to downcore samples.

Overall, relative total BHP concentrations increased in the upmarsh direction, particularly in downcore samples. However, in surface samples, relative total BHP concentrations decreased from the lower- to upper-marsh zones.

### BHP compounds

A total of 26, 18, and 17 distinct BHP compounds were identified in samples from DI, NBIN, and NBIS, respectively (Tables S1, S2, and S3). Despite variations in BHP diversity between sites, minimal differences were observed across marsh zones within each site.

The most abundant BHPs identified were BHT (and BHT isomer), aminotriol, BHT cyclitol ether, and methylated BHT, which was identified as 2Me-BHT, with adenosylhopane being prominent in DI samples. These dominant compounds collectively accounted for 86%–99%, 95%–99%, and 90%–98% of total BHP concentrations at DI, NBIN, and NBIS, respectively.

The classification of BHPs includes six major categories: anhydro, BHTs, amino, sugar, aminosugar, and soil marker BHPs. Among these, BHTs were consistently the most abundant across all three sites, followed by amino, aminosugar, and soil marker BHPs. Sugar BHPs were present in low abundance in DI samples and were entirely absent in samples from NBIN and NBIS.

#### BHP compound group: BHTs

**Figure 3.**
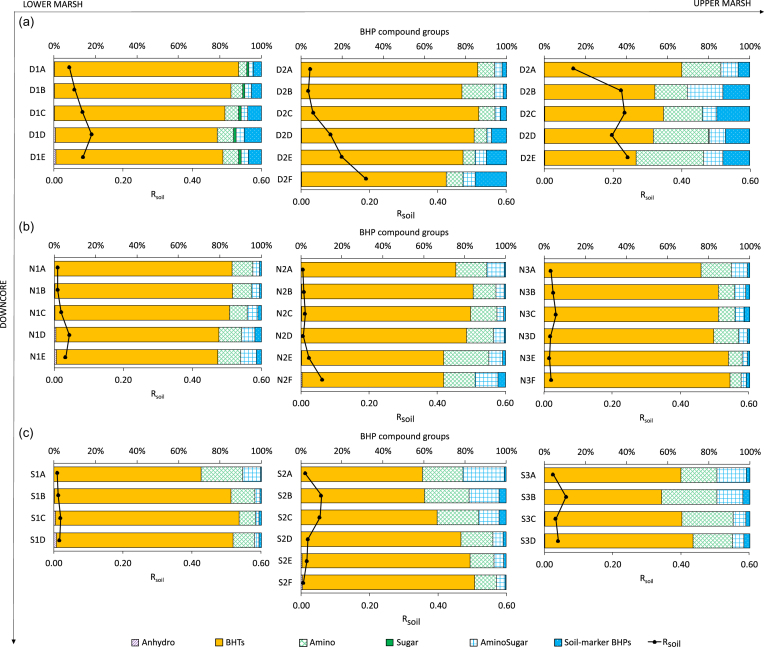
Downcore fractional abundance of BHPs and *R*_soil_ across cores from lower, mid and upper marsh areas at (a) DI, (b) NBI, north lagoon, and (c) NBI, south lagoon.

Across the three sites, BHT was the most abundant compound, contributing an average of 60.7%, 75.2%, and 69.0% of total BHPs at DI, NBIN, and NBIS, respectively (Fig. 3, Table 3). Its average relative concentration was highest in DI samples at 436 µg/g OC, followed by 403 µg/g OC in NBIS and 330 µg/g OC in NBIN (Tables S1, S2, and S3). The proportional abundance of BHT ranged from 40.8% to 82.0% at DI, 56.0% to 86.1% at NBIN, and 52.3% to 76.1% at NBIS. Downcore trends in BHT concentrations varied across sites. A decrease was observed in DI cores and in lower- and mid-marsh cores from NBIN, while concentrations increased downcore in NBIS cores and the NBIN upper-marsh core.

At the three sites, 2Me-BHT was also present, with average relative concentrations of 30 µg/g OC at DI, 22 µg/g OC at NBIN, and 31 µg/g OC at NBIS. This compound accounted for 4.2%, 5.0%, and 5.3% of total BHPs, respectively. Another notable compound, BHT isomer, was detected in all samples, with average relative concentrations of 11 µg/g OC at DI, 9 µg/g OC at NBIN, and 8 µg/g OC at NBIS. It accounted for 1.5%, 2.0%, and 1.4% of total BHPs, respectively. Some samples from DI showed presence of BHHexol.

#### BHP compound group: soil-marker BHPs

Six soil-marker (nucleoside) BHPs were detected in samples from DI, compared to only four at NBIN and three at NBIS. Soil-marker BHP relative concentrations generally increased both downcore and upmarsh. Adenosylhopane was the most abundant soil-marker BHP across all three sites, being present in every sample (Tables S1, S2, and S3). Its highest average relative concentration was observed at DI, at 41 µg/g OC, accounting for 5.7% of total BHPs. NBIN and NBIS show markedly lower average relative concentrations of adenosylhopane, at 5 µg/g OC (1.0% of total BHPs) and 4 µg/g OC (0.7% of total BHPs), respectively.

At DI, adenosylhopane Types 2 and 3 and all methylated homologues were widely present, whereas NBIN samples only contained a single methylated homologue (2Me-adenosylhopane Type 2). Samples from NBIS contained adenosylhopane Type 2 and its methylated homologue.

#### BHP compound group: amino-BHPs

The fractional abundance of amino-BHP compounds was higher in mid- and upper-marsh cores compared to those from the lower-marsh zone (Fig. 3, Table 3). Among the amino BHPs, aminotriol was the most abundant, present in all samples, with aminotetrol identified in some.

Aminotriol was the second most abundant BHP compound across the sites, averaging 103, 45, and 80 µg/g OC at DI, NBIN, and NBIS, respectively, accounting for 14.3%, 10.1%, and 13.7% of total BHPs (Tables S1, S2, and S3). Its fractional abundance increased upmarsh at all sites. Downcore distributions showed inverse correlations with BHT: fractional abundance of aminotriol increased downcore in DI and in the low- and mid-marsh zones of NBIN, while decreasing in NBIS cores and in the upper marsh core from NBIN.

Aminotetrol, a minor amino BHP, accounted for 0.7%, 0.4%, and 1.5% of total BHPs at DI, NBIN, and NBIS, respectively. Trace amounts of aminopentol were detected in a few samples. Methylated aminotriol was identified in all samples in trace amounts, while unsaturated aminopentol was present in a few samples across the sites.

#### BHP compound group: aminosugar-BHPs

The most abundant compound in the aminosugar group was BHT cyclitol ether, which showed average concentrations of 38, 20, and 36 µg/g OC in DI, NBIN, and NBIS samples, respectively (Tables S1, S2, and S3). This represented 5.3%, 4.6%, and 6.2% of total BHPs. Fractional abundance of BHT cyclitol ether increased upmarsh.

Other aminosugar compounds, such as BHT glucosamine, unsaturated BHtetra cyclitol ether, BHpenta cyclitol ether, and methylated BHpenta cyclitol ether, were detected in trace amounts in select samples.

#### BHP compound group: anhydro-BHPs

Trace amounts of anhydro-BHT was detected in some samples, constituting 0.3%, 0.3%, and 0.5% of total BHPs at DI, NBIN, and NBIS saltmarshes, respectively (Tables S1, S2, and S3). It was largely absent in surface sediments and cores from the upper marsh areas.

### *R*_soil_ index

*R*_soil_ index values ranged from 0.02 to 0.24 in samples from DI, and from 0.01 to 0.06 in samples from both NBIN and NBIS saltmarshes, having low values in the lower-marsh zone and corresponding with relatively low C:N ratio findings. At the NBI sites, there was a slight increase in *R*_soil_ from lower- to mid-marsh areas. At DI, *R*_soil_ values increased both downcore and upmarsh (Fig. 3, Table 3).

## Discussion

### OC content in DI and NBI saltmarshes

#### OC estimations and site-specific calibration

In this study, we compared two different methods (LoI and elemental analysis) for estimating OC in saltmarsh sediments. While LoI remains a widely used and accessible technique, our findings indicate that in the studied DI and NBI saltmarshes, LoI-based estimates may lead to a potential overestimation of OC compared to direct elemental analysis measurements. While the widely used equation (2) from Craft et al. (1991), produced reasonable estimates, it tended to overestimate OC in our sites. Therefore, where feasible, we recommend using elemental analysis to measure sedimentary carbon concentrations. In the absence of universal elemental analysis measurements, site-specific OC-LoI calibrations tailored to individual marshes may be usable if they produce robust relationships across a range of OC values. Although OC_EA_ and OC_LoI_ are interdependent—both relying on combusted samples to determine TIC—elemental analysis offers greater accuracy by reducing the uncertainties associated with OC estimates based on LoI conversion equations.

Tailored OC calibration equations for DI and NBI saltmarshes illustrate the need for site-specific approaches. The polynomial fit at DI indicates a non-linear relationship, with OC increasing as OM content increases. For NBIN, the stronger performance of the linear model suggests that a simple linear relationship best describes the OC–LoI relationship at this location. At NBIS, the polynomial model (8) better captures the curvature observed in the data, likely reflecting nonlinear controls on OC accumulation, such as increased autochthonous OC input from denser vegetation in surface samples from mid- and upper-marsh zones. This shape also closely mirrors the relationship described by Craft et al. (1991) (2), suggesting that equations in the style of (2) are effective for some sites.

These findings highlight the importance of bespoke calibrations for each saltmarsh. Generic models, such as those by Craft et al. (1991), may not accurately reflect the specific OC–LoI relationships of different sites. For instance, the deviation of equations (4) and (5) from Craft's et al. (1991) equation reinforces the necessity of tailored approaches when possible, and the preference for elemental analysis data. However, LoI remains a widely used and practical method for estimating OC in coastal sediments due to its accessibility and cost-effectiveness, especially in studies where elemental analysis is not available.

#### OC variations in DI and NBI saltmarshes

From here on, OC_EA_ is referred to as OC for simplicity. The concentrations of OC in DI and NBI saltmarshes exhibit clear patterns influenced by depth and marsh zone. The observed decline in OC with depth is likely due to higher biomass content near the surface, where recent vegetation growth contributes fresh organic material. With depth, this material diminishes as labile components decompose and undergo remineralization (Gore et al. 2024). These observations highlight the importance of analysing downcore samples, as surface OC values alone may not accurately reflect the depth-averaged concentrations within the sediment profile.

The transect trend of increasing OC from lower- to upper-marsh zones is consistent with findings from other studies (e.g. Smeaton et al. 2024) and is attributed to the greater vegetation density and diversity in upper-marsh areas. These factors enhance plant biomass inputs and stimulate microbial processes that drive OM accumulation and decomposition (Kim et al. 2022, Liu et al. 2024). The diminishing of this pattern with depth mirrors trends observed in other studies (e.g. Mueller et al. 2019, Kang et al. 2024).

Site-specific OC differences are likely due to local factors. The elevated OC levels at NBIS may result from its proximity to estuarine input (Grey et al. 2021), which enhances both sedimentation and OM accumulation through increased tidal exchange and terrestrial OM delivery compared to predominantly marine-fed systems (Miller et al. 2023, Smeaton et al. 2023, Peck et al. 2025). In contrast, NBIN had OC values almost two times lower compared to NBIS, which could be due to being fed by marine sources, as well as its young age. The high OC values at DI are likely linked to the older age of the marsh, which has allowed for a longer period of OM accumulation. This observation aligns with studies (e.g. Smeaton et al. 2024) that identify a positive relationship between marsh age and OC content. Our findings further support this relationship by showing that fresh, autochthonous OC dominates surface sediments but becomes increasingly degraded with depth (Fig. 2, Table 1). Despite this downcore degradation, the preserved OC appears to build up over time, likely due to reduced decomposition under sustained anoxic conditions and continued sediment burial.

#### C:N ratio: insights into the OC sources

The C:N ratio, when combined with other techniques, can provide insights into the origin of OM in coastal environments. Ratios between 4 and 15 are typically indicative of marine-derived OM, whereas values above 15 suggest terrestrial inputs (Meyers 1997). However, C:N ratios of soil OM can range around 10 (e.g. Hedges and Oades 1997, Tipping et al. 2016), complicating the interpretation of these values. Marsh vegetation such as *Spartina* species can have relatively high C:N ratios (31.1 ± 2.0) (Radabaugh et al. 2017, Lanari et al. 2018), meaning their contribution would generally elevate C:N.

Grey et al. (2021) reported a wide range of C:N ratios from 0.7 to 175.6 for saltmarsh areas at NBI. The highest C:N values (41.9 and 175.6) were associated with mudflat-saltmarsh transition zones north and south of the causeway, which had low nitrogen concentrations—likely due to denitrification in fine-grained, silt-rich sediments (Ye et al. 2024). These areas were not sampled in the present study. Excluding these two outliers, the remaining C:N ratios in Grey et al. (2021) ranged from 0.7 to 14.4, which are comparable to the values observed in this study. The lowest C:N values observed may reflect degradation and remineralization of OC, while the higher values likely indicate a greater contribution from terrestrial plant material.

### BHP distribution patterns and controls in saltmarshes

Previous coastal studies have focused on soil-marker BHPs to trace terrestrial OC along land-to-ocean transects (e.g. Doğrul Selver et al. 2012, 2015, Bischoff 2016) and to investigate soil development in deglaciating areas in the Arctic (Akhmetkaliyeva et al. 2025), but this is the first to assess their presence and distribution within saltmarsh sediments.

The observed correlation between absolute total BHP and OC concentrations underscores the dependence of BHP accumulation on carbon availability (Höfle et al. 2015). The more gradual decline in absolute total BHP concentrations compared to OC with depth, particularly at DI, likely reflects diagenetic changes over time, where more labile BHPs are degraded, and the more resistant ones persist in deeper sediments (Sinninghe Damsté et al. 1995, Drozd et al. 2023, Yin et al. 2024). This stabilization might represent a point where inputs and degradation reach some balance. In contrast, the continued variation in absolute total BHP concentrations in cores from NBIN and NBIS suggests that marsh age can influence BHP accumulation, with younger sediments undergoing ongoing degradation and having had less time for preservation processes to stabilize. Additionally, the upmarsh gradient in absolute total BHP concentrations, particularly pronounced in the top of the core samples, reflects the higher vegetation density and diversity in upper marsh areas, which enhance OM inputs (Pondell and Canuel 2022, Miller et al. 2023).

On the other hand, the elevated relative total BHP concentrations downcore in upper-marsh cores suggest that BHPs become an increasingly large component of OC composition over time. Similarly, high relative total BHP concentrations at DI and NBIS and greater BHP structural diversity at DI, suggests that relative total BHP concentrations vary less with depth beyond ∼30 cm, indicating a stabilization over time. This pattern suggests that much older marshes like DI may have developed greater microbial diversity, contributing to this structural diversity. NBI marshes have lower structural diversity, likely due to the younger age of the saltmarshes. Higher relative total BHP concentrations at NBIS compared to NBIN, may reflect a slightly longer accumulation period and higher freshwater input (Grey et al. 2021), which likely influence biomarker concentrations rather than long-term microbial community development.

The lower relative total BHP concentrations in surface samples from the upper marsh cores at DI and NBIN, and mid- and upper-marsh cores at NBIS, compared to deeper samples, may reflect the preferential degradation of more labile OM over time, resulting in a greater relative contribution of BHPs to the remaining OC in deeper sediments. The general upmarsh increase in relative total BHP concentrations, particularly in downcore samples, may reflect longer-term OM burial and preservation in older sediments, leading to BHP accumulation. In contrast, the decrease in relative total BHP concentrations from lower- to upper-marsh zones in surface samples likely reflects a smaller fraction of BHPs in OC relative to increasing contributions from plant-derived OM.

The observation of declining OC and rising relative total BHP concentrations in mid- and upper-marsh cores is consistent with bacterial decomposition of plant-derived labile OC over time. As this labile OC is decomposed, recalcitrant OC including BHPs become a more prominent fraction of the preserved organic compounds downcore (Gore et al. 2024).

However, it is important to note that BHP profiles are shaped not only by microbial degradation and input, but also by broader site-specific factors. For example, halophytic plant species can significantly influence microbial community structure and enzyme activity in saltmarsh soils (Chaudhary et al. 2018), while sediment type and vegetation cover affect rhizosphere fungal communities (d’Entremont et al. 2021). These findings underscore the importance of understanding the sources and composition of plant-derived OM when evaluating OC dynamics in saltmarshes. From here on, we discuss relative BHP concentrations.

### OC signatures in non-soil BHP compounds

This study represents the first application of BHP analysis to explore carbon provenance in saltmarsh environments. Some of the most abundant BHPs: BHT, aminotriol, and BHT cyclitol ether are known to originate from multiple environmental sources (Talbot and Farrimond 2007, Talbot et al. 2008). However, in this section, we attempt to better understand their specific origins.

The downcore decrease in fractional BHT abundance, which is often used as pseudo-marine marker, in DI cores and in lower- and mid-marsh cores from NBIN likely reflects the dominant marine OC input brought by tidal activity. Conversely, the increase downcore in NBIS cores and the NBIN upper marsh core suggests *in-situ* BHT production. An inverse relationship between BHT and aminotriol suggests that fluctuations in BHT might be influenced by variations in aminotriol concentrations.

While BHT cyclitol ether is produced by various bacteria (Cooke 2011), it is not specific to a single environment. Its reported abundance varies across studies, sometimes indicating marine origins (Pearson 2009) and at other times reflecting terrestrial sources (Zhu et al. 2011). In the present study, the upmarsh increase in the fractional abundance of BHT cyclitol ether suggests a predominantly terrestrial origin. This observation may imply that as the marsh transitions from lower to upper zones, the microbial community shifts to include more terrestrial bacteria capable of producing BHT cyclitol ether.

Some compounds can serve as a signal of degradation of other BHP compounds. The absence of anhydro-BHT in surface sediments and cores from the upper marsh areas could indicate limited production or preservation in these zones. Anhydro-BHT is thought to be the diagenetic product of BHT, adenosylhopane, and related compounds (Cooke et al. 2008a, Zhu et al. 2011). Elevated proportions of anhydro-BHT have been observed in deeper sediments (Cooke et al. 2008b). The presence of anhydro-BHT in downcore samples may suggest *in-situ* degradation of BHT, adenosylhopane, and related compounds over time. However, only trace amounts of anhydro-BHT were measured in this study.

### Microbial contributions to methane dynamics in saltmarshes

In addition to serving as indicators of terrestrial OM input, certain BHPs also reflect microbial processes such as methanotrophy and sulfate reduction. These microbial signatures are relevant to interpreting BHP distributions in saltmarsh sediments. Here, we examine the distributions of aminotetrol, aminotriol, and aminopentol to assess contributions from methanotrophs and sulfate-reducing bacteria to sedimentary BHP pools and their potential role in regulating CH_4_ dynamics in Irish saltmarshes.

Saltmarshes emit less CH_4_ than freshwater wetlands because their sulfate-rich environment favours sulfate-reducing bacteria, which outcompete methanogens, thereby suppressing CH_4_ production (Le Mer and Roger 2001, Poffenbarger et al. 2011, Capooci et al. 2024). Also, methanotrophic bacteria, which metabolize CH_4_ in saltmarshes, play a significant role in reducing CH_4_ emissions to the atmosphere (Le Mer and Roger 2001). However, some studies suggest that CH_4_ can still form in saltmarshes through methylotrophic methanogenesis, a process not inhibited by sulfate reduction (Capooci et al. 2024).

**Figure 4.**
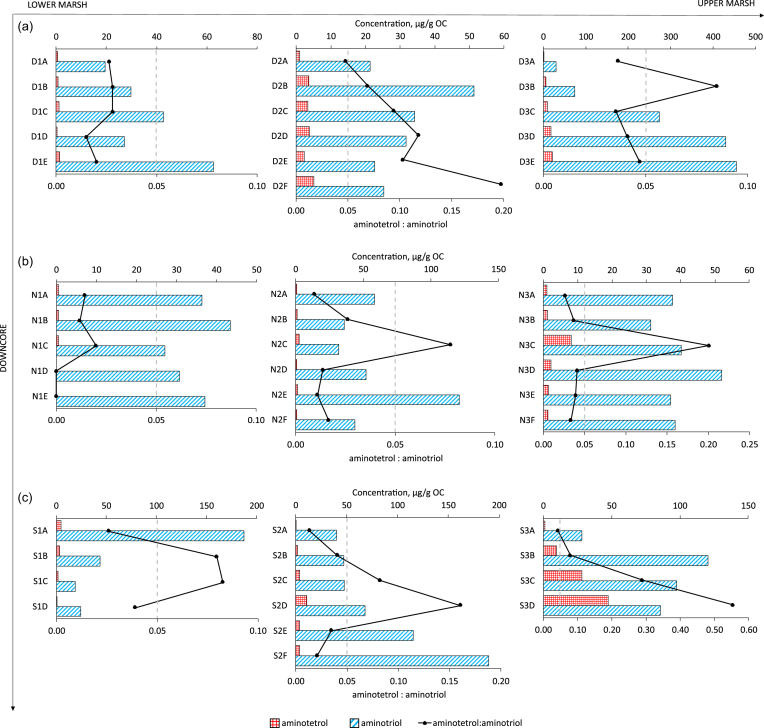
Downcore concentration of aminotriol and aminotetrol (µg/g OC), and aminotetrol: aminotriol relationship across cores from lower-, mid- and upper-marsh areas at (a) DI, (b) NBI, north lagoon, and (c) NBI, south lagoon.

Aminotetrol is produced by methanotrophs and some sulfate-reducing bacteria (*Desulfovibrio sp*., Blumenberg et al. 2006). The aminotetrol: aminotriol ratio helps identify the source of aminotetrol—ratios above 1:20 suggest methanotroph origins, while those below 1:20 indicate *Desulfovibrio* origins (Pytlak et al. 2021). Aminotetrol in lower- and upper-marsh zones at DI is largely *Desulfovibrio*-derived, whereas the mid-marsh core likely has methanotroph-derived aminotetrol, with the exception of the surface layer (Fig. 4a). At NBIN, aminotetrol is predominantly *Desulfovibrio*-derived (Fig. 4b). At NBIS, both methanotrophs and *Desulfovibrio* likely contribute, with methanotroph activity prevalent in the upper marsh core (excluding the top sample) and in mid-core sections of the lower- and mid-marsh zones (Fig. 4c).

Aminopentol is produced by Type I methane-oxidizing bacteria (Cvejic et al. 2000). In peatlands, Type I methanotrophs that produce aminopentol are found in surface soils, whereas deeper soils contain Type II methanotrophs, which produce aminotetrol and aminotriol (Esson et al. 2016, Rey-Sanchez et al. 2019). This pattern aligns with the presence of aminopentol in the top layers of lower-marsh cores at DI and NBIS. Its detection in mid-core samples suggests that Type I methanotroph activity can occur in saltmarsh sediments less affected by saltwater intrusion compared to lower-marsh zones.

The findings indicate that both methanotrophs and sulfate-reducing bacteria (*Desulfovibrio*) are present in Irish saltmarshes. Methanotroph activity is more pronounced in downcore samples, suggesting CH_4_ production from decomposing OM.

### Saltmarshes as transitional zones

The distribution of soil-specific BHP compounds provide insights into the development and ecological dynamics of saltmarshes. The higher number and concentration of soil-marker BHPs at DI are likely influenced by differences in sediment age across the sites. At DI, e.g. the top 20 cm of sediment in the lower marsh has accumulated over the past 100 years (Burke 2024), suggesting that deeper sediments, and those in the mid- and upper marsh, are likely older. In contrast, the NBI marshes are younger overall, having developed within the last two centuries (Grey et al. 2021), the deepest core being 54.5 cm deep.

Contrary to expectations, NBIS, despite its proximity to freshwater sources (Grey et al. 2021), exhibits low terrestrial signals similar to NBIN, suggesting a predominantly marine influence on sediment accumulation. This pattern may indicate that factors such as site age and bacterial activity within older, more stable sediments play a greater role in the accumulation of soil-marker BHPs. While we do not directly measure biomarker production, the observed distribution is consistent with potential *in-situ* synthesis of soil-marker BHPs under oxygen-limited conditions, as suggested by Kusch et al. (2021).

The broader distribution of adenosylhopane types and methylated homologues at DI, compared to the limited profiles at NBIN and NBIS, supports a potential influence of marsh age in shaping the accumulation and diversity of soil-marker BHPs, possibly through prolonged conditions favourable to *in-situ* production. However, further research is needed to fully understand the underlying mechanisms.

The general increase of soil-marker BHP relative concentrations both downcore and upmarsh likely reflects accumulation of these compounds in areas less influenced by tidal perturbation. This trend could result from increased inputs of soil-marker BHPs or their enhanced preservation compared to other structures (Zhu et al. 2011), or possible *in-situ* microbial production in older sediments (Kusch et al. 2021). In the latter case, soil-marker BHPs may not reflect direct terrestrial input, but rather *in-situ* production within the sediment matrix.

Despite the presence of adenosylhopane, and minor amounts of other soil-marker BHPs, indicating either some terrestrial input or *in-situ* production, BHP results indicate a significant influx of marine OC into the saltmarshes with the dominance of BHT reflected in the *R*_soil_ index. Near-zero *R*_soil_ index values in the lower-marsh zones, showing a predominance of marine-derived sedimentary OC, are consistent with other studies where marine-dominated environments were observed along river-ocean transects in offshore directions, and where low soil-marker BHP input (Zhu et al. 2011, Dogrul Selver et al. 2012, 2015) or *in-situ* production under marine conditions has also been documented (Kusch et al. 2021). Although originally developed for land-to-ocean transects, the *R*_soil_ index remains useful in saltmarsh settings, which, as transitional systems, integrate both marine and terrestrial inputs alongside substantial *in situ* production. Applying the proxy in this transitional context provides a valuable first-order assessment of sedimentary OC provenance.

### Sediment history, bacterial activity, and long-term OC dynamics

At the NBI sites, the *R*_soil_ index indicates that the sediment captured by the saltmarshes is predominantly marine in origin. The slight increase in *R*_soil_ from lower to mid-marsh suggests that a terrestrial signal strengthens upmarsh, but no significant pattern was observed downcore. This suggests that sedimentary OC has been predominantly marine in origin since the establishment of the saltmarsh. While some terrestrial OC is present throughout, the terrestrial signal did not become stronger, likely due to the relatively young age of the marshes. This finding challenges the expectation that NBIS would exhibit a more prominent terrestrial signal due to riverine input, a pattern observed in Scottish saltmarshes located along major rivers (Miller et al. 2023). Instead, it highlights that while the allochthonous OC captured by the saltmarsh primarily originates from marine sources, the age of the marsh also plays an important role in the bacterial signatures within its sediment.

At DI, the increase in *R*_soil_ index values both downcore and upmarsh indicate a predominance of marine-derived OC at the top of the core, potentially shifting towards a terrestrial signal. Although low, *R*_soil_ values in the top core samples indicate the presence of some terrestrial OC, suggesting that soil-marker BHP-producing bacteria are present alongside marine sediment. Given that the DI saltmarsh is older compared to the marshes at NBI, this shift is likely due to the long-term development of a bacterial community that favours soil production in the absence of regular saltwater inundation, creating aerobic or micro-oxic conditions. This interpretation aligns with the findings in Kusch et al. (2021), who reported soil-marker BHPs production in marine oxygen-deficient zones, challenging the assumption that these compounds exclusively reflect terrestrial input. Their study, along with the findings presented here, suggests that soil-specific biomarkers can be produced in a variety of environmental conditions, including redox and depositional settings such as older sediments, where such microbial communities may persist and contribute to biomarker signals.

The top 20 cm of cores in the pioneering-lower marsh zone at DI are ∼100 years old (Burke 2024). Although the exact age of sediments deeper than this, as well as those in the mid- and upper-marsh zones, remains uncertain, it is assumed that these sediments are significantly older. There is no known history of arable farming on the marsh, suggesting that the stronger terrestrial signal observed in older sediments is likely the result of long-term bacterial activity. Reduced interaction with incoming tidal water in downcore sediments and in the upper-marsh zone, which is flooded only a few times a year, likely leads to differences in microbial communities (Tebbe et al. 2022).

The decrease in OC concentrations with depth at DI suggests that microbial degradation of OC slows at greater depths, leaving behind more resistant forms of carbon that persist over time (Spivak et al. 2019, Gore et al. 2024). Our findings also indicate that the allochtonous OC is predominantly marine-derived, emphasizing the strong degree of connectivity between saltmarshes and the wider marine environment. In mid- and upper-marsh areas, a decrease in OC and an increase in total BHPs (µg/g OC) downcore demonstrate preservation of recalcitrant OC in older sediments, while young labile C at the top of the core decomposes. Although we do not have direct age constraints on the OC in our samples, our results are consistent with previous studies (Mueller et al. 2019, Houston et al. 2024), which suggest that younger, labile OC is more prone to degradation than older recalcitrant OC. These findings suggest similar mechanisms of preferential preservation may be at play in our system.

Although small amounts of soil-marker BHPs were detected in all surface samples, this suggests that they might have been introduced along with marine sediment. This could indicate that while soil bacteria may always be present in the environment, they require specific conditions, such as reduced saltwater intrusion and more aerobic or micro-oxic environment, to thrive and produce these markers in significant quantities. Evidence of similar ecological transitions has been reported by Dini-Andreote et al. (2016), who observed a shift from marine to terrestrial fungal communities over time, supporting the idea that microbial community composition can evolve significantly under prolonged isolation from marine influence.

Over long timescales, probably much more than 100 years, microbial communities likely shift towards bacteria capable of producing soil-marker BHPs. This shift, aligned with the increase in soil-marker BHP concentrations and *R*_soil_ values downcore and upmarsh, emphasizes the importance of time and environmental conditions in shaping the development of bacterial communities in saltmarshes.

### Study limitations, future recommendations, and policy implications

This study is the first to investigate OC sources in Irish saltmarshes using biomarkers (BHPs). While BHPs are effective tools for identifying the origin of sedimentary carbon, they have limitations in providing specific information on the origin of labile OC, such as that derived from plants or more easily degradable OM, which is critical for understanding long-term carbon sequestration. Moreover, while the *R*_soil_ proxy was developed to distinguish between terrestrial and marine BHP sources in land-to-ocean transects, its application to saltmarsh environments requires additional consideration. We apply *R*_soil_ here to explore broad source trends in saltmarsh sediments, while acknowledging that the proxy does not account for vascular plant-derived OM, which may be substantial. Therefore, it is recommended that future research incorporate complementary techniques, such as stable isotope analysis (e.g. *δ*^13^C, *δ*^15^N), to distinguish between these types of carbon and provide more accurate assessments of carbon storage potential. Autochthonous OC, originating within the ecosystem, is clearly countable as a contribution to climate regulation by sequestering atmospheric CO_2_. Allochthonous OC, however, can contribute as avoided emissions if it would have otherwise been released as CO_2_ (Mossman et al. 2024). Houston et al. (2024) argue that older, recalcitrant allochthonous carbon plays a key role in long-term OC preservation in saltmarshes when anoxic conditions are maintained, thus supporting its treatment as additional carbon, a conclusion that is also supported by this study. Further investigation of OC sources is essential to accurately assess the accountability of carbon sequestered by Irish saltmarshes.

Furthermore, the study’s findings highlight the dynamic nature of saltmarsh carbon storage. As observed in the DI site, there is evidence of a shift in microbial communities from marine to terrestrial with increasing age. This underlines the importance of considering the age and history of saltmarshes in carbon sequestration models. However, with rising sea levels, the likelihood of this transition may decrease, as prolonged tidal inundation could favour marine-associated microbial communities and influence carbon dynamics differently. Understanding these shifts in microbial activity over time, particularly in the context of changing sea levels and saltwater exposure, is critical for developing long-term strategies to conserve and restore saltmarshes as effective carbon sinks.

## Conclusions

In this novel study, Irish saltmarsh samples were taken from downcore sections of three lower- to upper-marsh transects to analyse OC concentrations, and sedimentary carbon sources via BHP biomarkers. The study found that OC concentrations generally decrease with depth, showing a significant drop below 30 cm, followed by stabilization at <1–2 wt%. This drop is likely due to the burial and decomposition of biomass that enters the marsh surface. BHP data and *R*_soil_ index values indicate that the majority of the sediment in all three marshes is of marine origin, but some soil-specific BHPs were also detected.

In the oldest transect, an increase in soil-marker BHPs and *R*_soil_ values deeper in the cores suggests a shift in the microbial community towards more terrestrial bacteria, likely due to less frequent tidal water inundation. These findings suggest that over time, saltmarshes may gradually transition to more terrestrial environments under certain conditions.

The allochthonous nature of sequestered sediments highlights the importance of maintaining connectivity to the marine environment. Tidal flow and sediment supply are critical for preserving saltmarsh function and ensuring their long-term role as carbon sinks. Effective conservation and management should focus on sustaining sediment supply and hydrological conditions to maximize their carbon storage potential.

## Supplementary Material

qvaf012_Supplemental_File

## Data Availability

The biomarker data underlying this article are available in the article and in its online supplementary. Raw biomarker data will be shared on reasonable request to the corresponding author.
