# Supplementary material for: Allochthonous carbon primarily of marine origin in Irish saltmarshes: novel insights from bacteriohopanepolyol biomarkers
Source: Sustain Microbiol. 2025 Jul 17;2(3):qvaf012. doi: 10.1093/sumbio/qvaf012 (PMC13375332; doi:10.1093/sumbio/qvaf012)
Supplement: Supplementary file 1 [file qvaf012_Supplemental_File.docx]

**Supplementary material**

Figure S1. BHP structures, adapted from Dogrul Selver 2015.

| Core structure | Head group | Name | Acetylated base peak ion(s) m/z |
| --- | --- | --- | --- |
| 1 | a | Bacteriohopanetetrol (BHT) | 655 |
| 2 | a | Methylated BHT | 669 |
| 1 | a’ | BHI isomer | 655 |
| 1 | b | Aminotriol | 714 |
| 2 | b | Methylated aminotriol | 728 |
| 1 | c | Aminotetrol | 772 |
| 1 | d | Aminopentol | 830 |
| 1 | e | 30-(5′-adenosyl)hopane | 746+788+830 |
| 1 | f | BHT cyclitol ether | 1,002+1,044 |
| 1 | g | N1-methylinosylhopane | 761 |
| 1 | g’ | Adenosylhopane_HG-Me_ | 802 |
| 1 | h | BHT glucosamine | 1,002 |
| 1 | i | Bacteriohopanehexol | 771 |

| LoI_450 | LoI_500 |
| --- | --- |
| 0.14 | 0.151061 |
| 0.12 | 0.137523 |
| 0.09 | 0.0922237 |
| 0.03 | 0.0266617 |
| 0.02 | 0.0176431 |
| 0.27 | 0.277933 |
| 0.21 | 0.226628 |
| 0.12 | 0.128218 |
| 0.05 | 0.0645138 |
| 0.06 | 0.0659409 |
| 0.08 | 0.104858 |
| 0.71 | 0.553592 |
| 0.23 | 0.246168 |
| 0.07 | 0.0534385 |
| 0.02 | 0.0162538 |
| 0.01 | 0.0103919 |
| 0.1 | 0.146713 |
| 0.06 | 0.113011 |
| 0.04 | 0.0724397 |
| 0.02 | 0.0280223 |
| 0.01 | 0.0195406 |
| 0.14 | 0.0929568 |
| 0.12 | 0.159968 |
| 0.15 | 0.179241 |
| 0.09 | 0.131202 |
| 0.02 | 0.0175398 |
| 0.01 | 0.0196033 |
| 0.27 | 0.343266 |
| 0.07 | 0.101451 |
| 0.04 | 0.0948082 |
| 0.07 | 0.177035 |
| 0.03 | 0.0732779 |
| 0.01 | 0.0219169 |
| 0.04 | 0.0694141 |
| 0.06 | 0.0869736 |
| 0.05 | 0.0765616 |
| 0.01 | 0.00251097 |
| 0.32 | 0.429448 |
| 0.35 | 0.415494 |
| 0.29 | 0.310719 |
| 0.09 | 0.113545 |
| 0 | 0.0103495 |
| 0.01 | 0.00897884 |
| 0.59 | 0.637255 |
| 0.15 | 0.15746 |
| 0.14 | 0.15482 |
| 0.01 | 0.0250425 |

Figure S2. Correlation plot of LoI data obtained using methods described in Howard (2014) (LoI_500) and Burke et al. (2022) (LoI_450).

Figure S3. Best-fit lines showing the relationship between OC measured via elemental analysis and LoI at 500 °C for 12 hours: (A1) linear trendline and (A2) polynomial trendline for Derrymore Island; (B1) linear trendline and (B2) polynomial trendline for NBI North Lagoon; (C1) linear trendline and (C2) polynomial trendline for NBI South Lagoon saltmarshes.

Table S1. Concentration of BHPs (µg/g OC) in downcore samples from the Derrymore Island saltmarsh. BDL – below detection limit.

| **Bacteriohopanepolyol compounds** | | **m/z** | **Lower Marsh** | | | | | **Mid-marsh** | | | | | | **Upper marsh** | | | | |
| --- | --- | --- | --- | --- | --- | --- | --- | --- | --- | --- | --- | --- | --- | --- | --- | --- | --- | --- |
|  |  |  | **D1A** | **D1B** | **D1C** | **D1D** | **D1E** | **D2A** | **D2B** | **D2C** | **D2D** | **D2E** | **D2F** | **D3A** | **D3B** | **D3C** | **D3D** | **D3E** |
| **Anhydro-BHT** | | 613 | 1 | 1 | 2 | 2 | 8 | BDL | BDL | 0.4 | 1 | 1 | 1 | BDL | BDL | 3 | 2 | 2 |
| **BHT** | BHT | 655 | 435 | 451 | 519 | 238 | 595 | 237 | 276 | 421 | 476 | 347 | 263 | 106 | 249 | 795 | 841 | 730 |
|  | BHT isomer | 655-II | 6 | 10 | 14 | 9 | 24 | 4 | 3 | 5 | 10 | 8 | 11 | 2 | 4 | 26 | 17 | 17 |
|  | 2Me-BHT | 669 | 26 | 38 | 57 | 31 | 74 | 10 | 13 | 21 | 24 | 19 | 10 | 7 | 40 | 40 | 33 | 37 |
|  | BHHexol | 771 | 3 | 3 | 4 | 2 | 5 | BDL | BDL | BDL | 3 | BDL | 1 | BDL | BDL | 18 | 22 | 11 |
| **Amino** | 35-aminotriol | 714 | 20 | 30 | 43 | 27 | 63 | 21 | 51 | 34 | 32 | 23 | 25 | 29 | 75 | 273 | 438 | 459 |
|  | Me-35-aminotriol | 728 | 1 | 1 | 2 | 1 | 2 | 1 | 4 | 3 | 2 | 4 | 2 | 2 | 6 | 6 | 10 | 15 |
|  | 35-aminotetrol | 772 | 1 | 1 | 1 | 0.4 | 1 | 1 | 4 | 3 | 4 | 2 | 5 | 1 | 6 | 10 | 18 | 22 |
|  | Unsaturated aminopentol | 828 | BDL | BDL | BDL | BDL | BDL | BDL | 0.3 | 0.2 | BDL | BDL | BDL | BDL | BDL | BDL | BDL | BDL |
|  | 35-aminopentol | 830 | 0.5 | 1 | 1 | BDL | BDL | BDL | BDL | BDL | BDL | BDL | BDL | BDL | BDL | BDL | BDL | BDL |
| **Sugar** | Tetra | 943+985 | 4 | 6 | 10 | 5 | 10 | BDL | BDL | BDL | BDL | BDL | BDL | BDL | BDL | BDL | BDL | BDL |
|  | Penta | 1001+1043 | BDL | BDL | BDL | BDL | BDL | BDL | BDL | BDL | BDL | BDL | BDL | BDL | BDL | BDL | 3 | BDL |
| **AminoSugar** | Unsaturated tetra | 1042 + 1000 | BDL | BDL | BDL | BDL | BDL | BDL | 0.4 | 0.5 | BDL | BDL | 1 | BDL | 1 | 3 | 3 | 5 |
|  | BHT cyclitol ether | 1002+1044 | 10 | 14 | 18 | 11 | 24 | 10 | 15 | 12 | 11 | 23 | 20 | 14 | 87 | 90 | 113 | 138 |
|  | BHT glucosamine | 1002 | 1 | 2 | 2 | 1 | 3 | 0.3 | BDL | 1 | 1 | BDL | BDL | BDL | BDL | BDL | 2 | 2 |
|  | Me-Tetra | 1016+1058 | BDL | 1 | 1 | 1 | 2 | BDL | BDL | BDL | BDL | BDL | BDL | BDL | BDL | BDL | BDL | 2 |
|  | Penta | 1102+1060 | 0.5 | 1 | 1 | 0.4 | 1 | 0.4 | 0.5 | 1 | 1 | 2 | 3 | 1 | 4 | 9 | 13 | 17 |
|  | Me-Penta | 1074+1116 | BDL | BDL | BDL | BDL | BDL | BDL | BDL | BDL | BDL | 1 | 1 | BDL | 1 | 2 | 2 | 2 |
|  | Hexa | 1160+1118 | 1 | 1 | 1 | BDL | BDL | BDL | BDL | BDL | BDL | BDL | BDL | BDL | BDL | 4 | 4 | 2 |
|  | Me-Hexa | 1174+1132 | 0.5 | 1 | 1 | BDL | BDL | BDL | BDL | BDL | BDL | BDL | BDL | BDL | BDL | BDL | BDL | BDL |
| **Adenosylhopanes** | Adenosylhopane | 746+788+830 | 15 | 22 | 37 | 25 | 41 | 6 | 5 | 12 | 32 | 22 | 17 | 5 | 18 | 125 | 110 | 160 |
|  | 2Me-adenosylhopane | 760+802+844 | BDL | BDL | BDL | BDL | 2 | BDL | BDL | BDL | BDL | BDL | 4 | 3 | 8 | 20 | 20 | 28 |
|  | Adenosylhopane Type 2 | 761 | 1 | 1 | 2 | 1 | BDL | BDL | BDL | 1 | 2 | 2 | 1 | 0.3 | 1 | 5 | 13 | 6 |
|  | 2Me-adenosylhopane Type 2 | 775 | 1 | 1 | 1 | BDL | BDL | BDL | 1 | 2 | 4 | 5 | 2 | 0.3 | 2 | 10 | 27 | 11 |
|  | Adenosylhopane Type 3 | 802 | 3 | 4 | 6 | 4 | 11 | BDL | BDL | BDL | 6 | 14 | 28 | 1 | 38 | 74 | 31 | 24 |
|  | 2Me-adenosylhopane Type 3 | 816 | 0.3 | 1 | 1 | BDL | 1 | BDL | BDL | BDL | 1 | 4 | 10 | 0.1 | 6 | 8 | 7 | 6 |
| **Σ BHPs** | | | 531 | 589 | 722 | 358 | 866 | 292 | 373 | 517 | 609 | 477 | 405 | 172 | 545 | 1522 | 1728 | 1788 |

Table S2. Concentration of BHPs (µg/g OC) in downcore samples from the North Bull Island, North lagoon saltmarsh. BDL – below detection limit.

| **Bacteriohopanepolyol compounds** | | **m/z** | **Lower marsh** | | | | | **Mid-marsh** | | | | | | **Upper marsh** | | | | | |
| --- | --- | --- | --- | --- | --- | --- | --- | --- | --- | --- | --- | --- | --- | --- | --- | --- | --- | --- | --- |
|  |  |  | **N1A** | **N1B** | **N1C** | **N1D** | **N1E** | **N2A** | **N2B** | **N2C** | **N2D** | **N2E** | **N2F** | **N3A** | **N3B** | **N3C** | **N3D** | **N3E** | **N3F** |
| **Anhydro-BHT** | | 613 | 1 | 1 | 1 | 2 | 3 | 1 | 1 | 1 | 1 | 1 | 2 | BDL | BDL | 1 | BDL | BDL | BDL |
| **BHT** | BHT | 655 | 316 | 386 | 245 | 187 | 229 | 303 | 282 | 223 | 323 | 329 | 166 | 201 | 366 | 529 | 345 | 501 | 676 |
|  | BHT isomer | 655-II | 5 | 8 | 11 | 13 | 13 | 3 | 3 | 2 | 5 | 25 | 15 | 7 | 5 | 7 | 4 | 7 | 16 |
|  | 2Me-BHT | 669 | 9 | 20 | 18 | 27 | 27 | 9 | 19 | 16 | 18 | 34 | 23 | 7 | 26 | 30 | 29 | 44 | 17 |
| **Amino** | 35-aminotriol | 714 | 36 | 43 | 27 | 31 | 38 | 59 | 37 | 32 | 52 | 121 | 44 | 38 | 31 | 41 | 51 | 37 | 39 |
|  | Me-35-aminotriol | 728 | 2 | 1 | 1 | 1 | 1 | 2 | 3 | 3 | 3 | 2 | 1 | 2 | 4 | 3 | 2 | 3 | 2 |
|  | 35-aminotetrol | 772 | 1 | 1 | 1 | BDL | BDL | 1 | 1 | 3 | 1 | 1 | 1 | 1 | 1 | 8 | 2 | 1 | 1 |
|  | Unsaturated aminopentol | 828 | BDL | BDL | BDL | BDL | BDL | 2 | BDL | BDL | 0.4 | BDL | BDL | BDL | BDL | BDL | 0.3 | BDL | BDL |
|  | 35-aminopentol | 830 | BDL | BDL | BDL | BDL | BDL | BDL | BDL | BDL | BDL | BDL | BDL | BDL | BDL | 1 | 0.3 | BDL | BDL |
| **AminoSugar** | Unsaturated tetra | 1042 + 1000 | BDL | BDL | BDL | BDL | BDL | 1 | 1 | 1 | 0.4 | BDL | 4 | 0.3 | 1 | 1 | 0.5 | 0.3 | BDL |
|  | BHT cyclitol ether | 1002+1044 | 12 | 15 | 14 | 17 | 23 | 35 | 14 | 9 | 22 | 35 | 25 | 20 | 24 | 28 | 17 | 13 | 19 |
|  | BHT glucosamine | 1002 | 1 | 2 | 1 | BDL | 2 | BDL | BDL | BDL | BDL | 1 | 1 | BDL | BDL | BDL | 1 | 0.5 | 1 |
|  | Me-Tetra | 1016+1058 | BDL | 0.3 | 0.3 | 1 | 1 | BDL | BDL | BDL | BDL | BDL | 1 | BDL | BDL | BDL | BDL | 1 | BDL |
|  | Penta | 1102+1060 | BDL | 0.3 | 1 | 1 | BDL | BDL | BDL | BDL | 0.4 | 1 | 1 | 1 | 1 | 1 | 1 | 0.3 | BDL |
| **Adenosylhopanes** | Adenosylhopane | 746+788+830 | 3 | 4 | 4 | 6 | 6 | 2 | 3 | 2 | 2 | 6 | 8 | 2 | 4 | 12 | 2 | 3 | 9 |
|  | Adenosylhopane Type 2 | 761 | BDL | BDL | 1 | 2 | 2 | BDL | BDL | 0.4 | BDL | 2 | 2 | 1 | 2 | 2 | 1 | 1 | 1 |
|  | 2Me-adenosylhopane Type 2 | 775 | BDL | BDL | BDL | 0.3 | BDL | BDL | BDL | 1 | 0.4 | 1 | 1 | 1 | 3 | 5 | 3 | 3 | 3 |
|  | Adenosylhopane Type 3 | 802 | BDL | BDL | BDL | 1 | BDL | BDL | BDL | BDL | BDL | BDL | 1 | BDL | BDL | BDL | BDL | BDL | BDL |
| **Σ BHPs** | | | 385 | 482 | 325 | 288 | 345 | 418 | 363 | 292 | 427 | 559 | 296 | 281 | 468 | 669 | 458 | 616 | 786 |

Table S3. Concentration of BHPs (µg/g OC) in downcore samples from the North Bull Island, South lagoon saltmarsh. BDL – below detection limit.

| **Bacteriohopanepolyol compounds** | | **m/z** | **Lower marsh** | | | | **Mid-marsh** | | | | | | **Upper marsh** | | | |
| --- | --- | --- | --- | --- | --- | --- | --- | --- | --- | --- | --- | --- | --- | --- | --- | --- |
|  |  |  | **S1A** | **S1B** | **S1C** | **S1D** | **S2A** | **S2B** | **S2C** | **S2D** | **S2E** | **S2F** | **S3A** | **S3B** | **S3C** | **S3D** |
| **Anhydro-BHT** | | 613 | BDL | 1 | 2 | 3 | BDL | 0.1 | 0.3 | 1 | 6 | 15 | BDL | BDL | 1 | 2 |
| **BHT** | BHT | 655 | 714 | 294 | 207 | 184 | 123 | 127 | 158 | 379 | 785 | 1469 | 113 | 269 | 311 | 509 |
|  | BHT isomer | 655-II | 8 | 4 | 7 | 9 | 1 | 3 | 2 | 3 | 20 | 45 | 2 | 4 | 3 | 5 |
|  | 2Me-BHT | 669 | 24 | 63 | 37 | 17 | 3 | 12 | 19 | 36 | 46 | 106 | 4 | 20 | 29 | 17 |
| **Amino** | 35-aminotriol | 714 | 190 | 44 | 19 | 25 | 40 | 47 | 49 | 68 | 114 | 192 | 28 | 123 | 96 | 87 |
|  | Me-35-aminotriol | 728 | 4 | 1 | 1 | BDL | 2 | 2 | 2 | 3 | 5 | 7 | 2 | 6 | 4 | 7 |
|  | 35-aminotetrol | 772 | 5 | 4 | 2 | 1 | 1 | 2 | 4 | 11 | 4 | 4 | 1 | 10 | 28 | 48 |
|  | Unsaturated aminopentol | 828 | 5 | BDL | BDL | BDL | BDL | 0.3 | 0.5 | 0.5 | BDL | BDL | BDL | BDL | BDL | BDL |
|  | 35-aminopentol | 830 | 5 | BDL | BDL | BDL | BDL | BDL | BDL | BDL | BDL | BDL | BDL | BDL | BDL | BDL |
| **AminoSugar** | Unsaturated tetra | 1042 + 1000 | 2 | 0.3 | 0.2 | 0.4 | 1 | 1 | 0.5 | 1 | BDL | BDL | 0.4 | BDL | 1 | BDL |
|  | BHT cyclitol ether | 1002+1044 | 86 | 9 | 4 | 5 | 43 | 34 | 26 | 25 | 44 | 77 | 25 | 62 | 29 | 37 |
|  | BHT glucosamine | 1002 | BDL | BDL | BDL | BDL | BDL | BDL | BDL | BDL | 2 | 5 | BDL | BDL | BDL | BDL |
|  | Penta | 1102+1060 | 1 | 1 | 0.3 | BDL | 0.4 | 1 | 1 | 1 | 1 | BDL | 1 | 2 | 2 | 2 |
|  | Me-Penta | 1074+1116 | BDL | BDL | BDL | BDL | BDL | BDL | BDL | BDL | BDL | BDL | BDL | 1 | BDL | BDL |
| **Adenosylhopanes** | Adenosylhopane | 746+788+830 | 4 | 3 | 3 | 3 | 1 | 2 | 1 | 3 | 8 | 7 | 1 | 5 | 4 | 9 |
|  | Adenosylhopane Type 2 | 761 | 1 | BDL | BDL | BDL | BDL | 2 | 2 | 2 | 3 | 2 | 1 | 4 | 2 | 4 |
|  | 2Me-adenosylhopane Type 2 | 775 | 1 | 1 | 1 | BDL | BDL | 4 | 6 | 4 | 3 | 2 | 1 | 9 | 4 | 9 |
| **Σ BHPs** | | | 1050 | 424 | 284 | 247 | 214 | 238 | 270 | 538 | 1042 | 1931 | 179 | 513 | 514 | 737 |
